# Hepatotoxicity in patients with non-small cell lung cancer treated with sotorasib after prior immunotherapy: a comprehensive clinical and pharmacokinetic analysis

**DOI:** 10.1016/j.ebiom.2024.105074

**Published:** 2024-03-19

**Authors:** Sophie M. Ernst, Maaike M. Hofman, Tessa E. van der Horst, Marthe S. Paats, Frank W.J. Heijboer, Joachim G.J.V. Aerts, Daphne W. Dumoulin, Robin Cornelissen, Jan H. von der Thüsen, Peter de Bruijn, Esther Oomen-de Hoop, Ron H.J. Mathijssen, Stijn L.W. Koolen, Anne-Marie C. Dingemans

**Affiliations:** a Department of Respiratory Medicine, Erasmus MC Cancer Institute University Medical Center, Doctor Molewaterplein 40, Rotterdam 3015 GD, the Netherlands; b Department of Medical Oncology, Erasmus MC Cancer Institute University Medical Center, Doctor Molewaterplein 40, Rotterdam 3015 GD, the Netherlands; c Department of Pathology, Erasmus University Medical Center, Doctor Molewaterplein 40, Rotterdam 3015 GD, the Netherlands; d Department of Pharmacy, Erasmus University Medical Center, Doctor Molewaterplein 40, Rotterdam 3015 GD, the Netherlands

**Keywords:** NSCLC, KRAS, Sotorasib, Hepatitis, Immunotherapy

## Abstract

**Background:**

Sotorasib given after immunotherapy could put patients at increased risk of hepatotoxicity. Therefore, there is a need to gain insight into the potential correlation between anti-PD-(L)1 treatment, anti-PD-(L)1 concentrations, sotorasib concentrations, and the incidence of hepatotoxicity during sotorasib.

**Methods:**

Patients with *KRAS*^G12C^-mutated NSCLC treated with sotorasib were prospectively enrolled in our biomarker cohort study (NCT05221372). Plasma samples were collected prior and during sotorasib treatment for anti-PD-1 and sotorasib concentrations. ALT/AST/ALP/GGT increases were collected prospectively and graded according to CTCAEv5.0. Severe hepatotoxicity was defined as grade ≥3 ALT/AST/ALP/GGT increase.

**Findings:**

Of the 91 included patients, 80 (88%) received prior anti-PD-(L)1. Prior anti-PD-(L)1 and prior immune-related hepatotoxicity were associated with a higher incidence of severe hepatotoxicity (35% versus 0%, *p* = 0.016 and 75% versus 31%, *p* = 0.019, respectively). Patients with an interval of ≤6 weeks between anti-PD-(L)1 and sotorasib (*n* = 18) had a significantly higher incidence of severe hepatotoxicity than those with a 6–12 week (*n* = 24) and ≥12 week (*n* = 38) interval (83% versus 33% versus 13%, respectively, *p* < 0.0001). Sotorasib trough concentrations did not differ significantly between those with or without severe hepatotoxicity (106 versus 126 ng/mL, *p* = 0.16). Pembrolizumab concentrations were higher in those with severe hepatotoxicity versus those without (25.6 versus 6.1 μg/mL, *p* < 0.0001).

**Interpretation:**

In this preliminary prospective study, sotorasib after PD-(L)1 blockade was associated with severe hepatotoxicity, especially in patients with a short interval between treatments, prior immune-related hepatitis and higher anti-PD-1 plasma concentrations. Our results suggest a minimum interval of 6 weeks between anti-PD-(L)1 and sotorasib to minimize the risk of hepatotoxicity.

**Funding:**

None.

**Research in context**

**Evidence before this study**

We searched PubMed for clinical trials, observational studies and case reports from January 1st, 2021 to October 17th, 2023, using the search terms “sotorasib”, “hepatitis”, “hepatotoxicity” and “liver”. We found that there is some prior evidence of sotorasib-related hepatotoxicity after immunotherapy. However, these studies have been limited by their retrospective nature, and more importantly, they do not investigate the potential influence of systemic sotorasib exposure or of anti-PD-(L)1 plasma concentrations on the development of severe hepatotoxicity.

**Added value of this study**

In this prospective real-world study we incorporated anti-PD-1 and sotorasib plasma concentrations to provide a comprehensive clinical and pharmacokinetic analysis of the time-dependent relationship between sequential anti-PD-(L)1 and sotorasib treatment and the development of severe hepatotoxicity. Our study found that PD-(L)1 blockade followed by sotorasib was associated with severe hepatotoxicity, especially in patients with a short interval between the two treatments and in patients who experienced immune-related hepatitis during prior anti-PD-(L)1 treatment. Importantly, patients with higher pembrolizumab plasma concentrations were more susceptible to developing severe hepatotoxicity than those with lower plasma concentrations. Sotorasib trough concentrations did not differ between those with or without severe hepatotoxicity, which further suggests the influence of prior anti-PD-(L)1 treatment.

**Implications of all the available evidence**

This study suggests that severe hepatotoxicity is a significant concern in patients receiving sequential anti-PD-(L)1 followed by sotorasib, especially in those with a short interval between treatments, prior immune-related hepatitis or higher pembrolizumab plasma concentrations. These data suggest a minimum interval of six weeks between the last anti-PD-(L)1 course and sotorasib initiation to reduce the risk of hepatotoxicity. However, considering the limitations that are inherent to the limited sample size, these findings should be considered preliminary. Larger, randomized trials are needed to validate and better understand these associations.

## Introduction

The treatment paradigm for patients with non-small cell lung cancer (NSCLC) has changed dramatically over the past decades with the emergence of targeted therapies for oncogenic driver alterations and immunotherapy.^1^, ^2^, ^3^, ^4^ Among Western populations, Kirsten rat sarcoma viral oncogene homolog (*KRAS*) is the most common oncogenic driver alteration in non-squamous NSCLC.^5^ Until recently, attempts to directly or indirectly target *KRAS* or its downstream signalling have been unsuccessful. However, the long-awaited *KRAS*^G12C^-specific inhibitors have now made their entrance. In the phase 3 CodeBreaK 200 trial, sotorasib demonstrated a statistically significant improvement in progression-free survival (PFS) compared to docetaxel with a favourable toxicity profile.^4^ Sotorasib has now received FDA and EMA approval for adult patients with pre-treated advanced or metastatic *KRAS*^G12C^-mutated NSCLC.

However, now that sotorasib is making its way to daily clinical practice, there is growing evidence that sotorasib after prior anti-PD-(L)1 therapy might put patients at a higher risk of immune-related toxicities.^6^, ^7^, ^8^, ^9^ For instance, the CodeBreaK 100/101 phase 1 b dose exploration showed a higher incidence of grade 3 and 4 treatment-related adverse events, predominantly hepatotoxicity, in patients treated with sotorasib in combination with pembrolizumab or atezolizumab concurrently compared to those receiving a lead–in with sotorasib (74% versus 53% for combination with pembrolizumab and 50% versus 30% for combination with atezolizumab).^10^ A French retrospective cohort study also showed that sequential anti-PD-(L)1 and sotorasib treatment was associated with an increased risk of severe hepatotoxicity: 33% of the patients who received anti–PD-(L)1 as the last line of treatment before sotorasib initiation experienced severe hepatotoxicity, versus 11% of patients who received anti–PD-(L)1 followed by at least one treatment regimen before sotorasib.^6^ However, this study was limited by its retrospective nature. Furthermore, there was a lack of data regarding the correlation between systemic sotorasib exposure and toxicity, as well as the relationship between toxicity during sotorasib treatment and plasma concentrations of anti-PD-(L)1 agents. As the majority of patients who are eligible for treatment with sotorasib have received upfront anti-PD-(L)1 treatment, there is a strong need to shed light on these aspects that could play a crucial role in understanding and addressing the risk of hepatotoxicity in this patient population. Moreover, considering the FDA’s post-marketing requirement to compare sotorasib 960 mg–240 mg daily (NCT04933695), there is particular interest in data on systemic sotorasib plasma concentrations.

In this study, we investigated a large prospective cohort of patients with *KRAS*^G12C^-mutated NSCLC treated with sotorasib to explore the relationship between prior anti-PD-(L)1 therapy and severe hepatotoxicity during sotorasib treatment. Furthermore, we investigated the relationship between anti-PD-1 and sotorasib plasma concentrations and the occurrence of severe hepatotoxicity.

## Methods

### Study design

All patients with *KRAS*^G12C^-mutated NSCLC who were eligible for sotorasib were prospectively included in our START-TKI biomarker study (NCT05221372). The START-TKI study is a prospective, observational multicentre cohort study in which additional blood samples are collected for circulating tumour DNA and pharmacokinetic analysis during the standard outpatient visits of patients with oncogene-driven advanced NSCLC for which they receive treatment with tyrosine kinase inhibitors or small molecules. Demographic and clinical data, including age at diagnosis, sex (self-reported by patient), type of previous anti-PD-(L)1 treatment, date of first and last anti-PD-(L)1 infusion, and prior immune-related toxicities, were collected from the patient records at baseline. In case of several previous lines of anti-PD-(L)1 treatment, the most recent treatment line before sotorasib initiation was considered. Adverse events (AEs) were prospectively reviewed at every outpatient visit and graded according to the Common Terminology Criteria for Adverse Events (CTCAE) Version 5. Hepatotoxicity was defined as increase in alanine aminotransferase (ALT), aspartate transferase (AST), alkaline phosphatase (ALP) or gamma glutamyltransferase (GGT). Severe hepatotoxicity was defined as CTCAE grade ≥3 increase. Causality between the AEs and sotorasib were determined by the treating physician. Patients who discontinued sotorasib within 4 weeks after treatment start were excluded from this analysis, unless they discontinued sotorasib because of hepatotoxicity, to ensure adequate time on treatment for the potential development of hepatotoxicity. PFS was defined as the time from sotorasib initiation until disease progression as assessed per RECIST version 1.1, or death from any cause. Patients who had not progressed at the time of cut-off or had discontinued treatment for a reason other than disease progression were censored at the date of the last response evaluation per CT scan without disease progression. Overall survival (OS) was defined as the time from sotorasib initiation until death of any cause. Patients who were still alive at data cut-off were censored at the date they were last known to be alive. Overall response rate (ORR) was assessed per RECIST version 1.1. Sotorasib was supplied by Amgen (Thousand Oaks, CA, United States) via the named patient program and post-approval access program that was available in the Netherlands until March 31st, 2023. Sotorasib was prescribed according to the prescribing information with a recommended dose of 960 mg orally once daily. Dose reductions were also done according to the prescribing information (first dose reduction level: 480 mg; second reduction dose level: 240 mg).^11^

### Exploratory plasma drug concentrations

Blood samples were drawn prior to treatment start and at every outpatient visit thereafter until sotorasib discontinuation. A 4.0 mL lithium-heparin tube was collected for plasma drug concentration analysis. Plasma anti-PD-1 concentrations were measured in samples that were taken prior to the start of sotorasib. In case of a missing pre-treatment sample, the first subsequent sample during sotorasib treatment was used for anti-PD-1 concentration analysis. Plasma sotorasib concentrations were measured in all samples taken during the outpatient visits for the entire treatment duration. The first plasma sample for sotorasib concentration analysis was taken after steady state of sotorasib had been reached (22 days), generally 4–5 weeks after treatment initiation.^11^ Patients were asked to delay their sotorasib intake until after venipuncture and were asked the date and time of their last intake prior to venipuncture. This was used to calculate the time in hours between the venipuncture and the time of next sotorasib intake (24 h after last intake).

Sotorasib plasma concentrations were measured by validated liquid chromatography-mass spectrometry. Sotorasib was quantitated by using an ultra-performance liquid chromatography tandem mass spectrometry (UPLC-MS/MS) method. Aliquots of 25 μL of human plasma samples for the quantitation of sotorasib were deproteinized after the addition of 100 μL of Internal Standard Solution (erlotinib-d6 in acetonitrile). After vigorously vortex mixing for 5 s and centrifugation for 10 min at 18,000∗g, 50 μL of the clear supernatant was transferred to a 96-well plate after which 100 μL of water/formic acid/ammonium formate (100:0.1:0.02, v/v/v) was added. After shaking for 5 min on a rocking platform, aliquots of 2 μL were injected into the UPLC-MS/MS system. The column effluent was monitored using the multiple reaction monitoring mode (MRM). Weighted (1/concentration^2^) linear regression analysis for sotorasib of peak area ratios of analyte and Internal Standard, versus concentration of analyte were used for the quantitation. Peak area ratios of analytes versus the Internal Standard were a function of the concentration from 20.0 to 2000 ng/mL, with the LOQ validated at a concentration of 20.0 ng/mL. The within- and between-run precisions at four tested concentrations, including the LOQ, were ≤2.39 and ≤ 2.48%, respectively, while the average accuracy ranged from 93.0 to 100.3%.

The sotorasib concentration prior to the next dose of sotorasib, defined as the trough concentration (C_trough_), was extrapolated with the following equation^12^:

$$C_{trough}=C_{sample}*e^{\left(-T*\;\frac{0.693}{T_{half}}\right)}$$

C_sample_ is the concentration in the plasma sample, -*T* the time in hours between the venipuncture and the next dose of sotorasib and T_half_ the half-life of sotorasib (5 h). C_trough_ which were extrapolated at more than twice the half-life of sotorasib were excluded from statistical analyses as they were considered insufficiently reliable, but were used in visualizations of plasma concentrations. The C_trough_ per sample was used to calculate the median plasma concentration per patient. In patients with severe hepatotoxicity, the samples from the treatment period until the onset of severe hepatotoxicity were used for analysis. In patients without severe hepatotoxicity the samples throughout the entire treatment period were used. The median C_trough_ was assessed for the different dose levels (960 mg versus lower doses) by analysing the samples collected during the administration of the corresponding doses.

The monoclonal antibodies (mAB) nivolumab and pembrolizumab were quantitated by using the mABXmise monoclonal antibodies quantification kit multiplex (Promise Proteomics, France; catalogue number OTDM1-RUO) which is based on the stable isotope dilution coupled to mass spectrometry analysis. The internal standard is a Stable-Isotopically-Labelled mAb (SIL-mAb), with a sequence highly similar to the one of the targeted mAb and coated at same amount in each well of the 96 well-plate. Calibration curves for nivolumab and pembrolizumab were linear in the range of 2.00 μg/mL to 100 μg/m, with a lower limit of quantitation of 2.00 μg/mL.^13^

### Ethics

The study was approved by the medical ethical committee of the Erasmus Medical Center, Rotterdam, The Netherlands (MEC 16–643), and is conducted in accordance with good clinical practice guidelines and the Declaration of Helsinki’s ethical principles for medical research. All patients provided written informed consent prior to any study procedures and data collection.

### Statistics

Statistical analysis was performed with IBM SPSS version 25.0 software. Categorical characteristics were compared using Fisher’s Exact test (in case of an expected cell frequency of <5) or Chi–Square test, differences in continuous data were compared by Student’s T-test, Mann–Whitney U-test or Kruskal–Wallis test. Odds ratios (OR) were calculated and Haldane correction was applied where appropriate. Univariate logistic regression analysis was performed to identify variables associated with severe hepatotoxicity. Prior to analysis, linearity was assessed by Box–Tidwell test and in case of non-linearity the respective variables were transformed into dichotomous variables. Due to the modest sample size, subsequent multivariable logistic regression analysis was not performed. Median follow-up duration with corresponding 95% confidence intervals (CI) were calculated by reverse Kaplan–Meier methodology. For survival analysis, both the origin and start times were set as the date of the initiation of sotorasib. PFS and OS were estimated by Kaplan–Meier methodology and compared by log-rank test. Univariate cox proportional hazards regression was used to estimate the hazard ratios (HR). The Agresti-Coull method was used to estimate 95% CIs for the ORR. Correlations were analysed with the Spearman’s rho test. Receiver Operating Characteristic (ROC) curve analysis was used to explore the optimal cut-off value of pembrolizumab plasma concentrations, to aid in predicting the occurrence of severe hepatotoxicity. The optimal cut-off value was assessed by Youden index. The Agresti-Coull method was used to estimate 95% CIs for the sensitivity, specificity, positive predictive value and negative predictive value of the chosen cut-off value. ROC curve analysis was not performed for nivolumab concentrations due to the limited sample size. All analyses were two-sided and *p*-values <0.05 were considered statistically significant. R statistical software version 4.3.1 was used for visualizations of the sotorasib plasma concentrations.

### Role of funders

None.

## Results

Ninety-one patients with *KRAS*^G12C^-mutated NSCLC who started treatment with sotorasib between March 2021 and April 2023 were included. At data cut-off (May 26th 2023), median follow-up duration was 12.1 months (95% CI 7.8–16.4) and median time on sotorasib was 3.7 months (range 0.9–25.6).

The clinical characteristics of the cohort are summarized in Table 1. Eighty patients (88%) had received prior anti-PD-(L)1 treatment, of whom 51 (64%) received anti–PD-(L)1 treatment as last line of treatment before sotorasib initiation. Median time between the last course of anti-PD-(L)1 and sotorasib initiation was 62 days (range 16–1353). A total of 58 patients (73%) received pembrolizumab as monotherapy or in combination with platinum-based chemotherapy, 11 received nivolumab, nine durvalumab and two atezolizumab. Eight patients had experienced immune-related hepatitis during prior anti-PD-(L)1 treatment, of which three patients had discontinued anti-PD-(L)1 treatment due to the immune-related hepatitis. All eight patients were off steroids and their liver enzymes had normalized prior to sotorasib initiation. Of the 11 patients who did not receive prior anti-PD-(L)1 treatment, one patient was treatment naïve and ten patients had received platinum-based chemotherapy prior to start of sotorasib.

**Table 1:** Demographic and clinical characteristics of the patients who received prior anti-PD-(L)1 treatment and the entire cohort.

	Prior anti-PD-(L)1 *n* = 80	No prior anti-PD-(L)1 *n* = 11	Entire cohort *N* = 91
Age (years), median (IQR)	63 (57–70)	70 (64–75)	64 (58–70)
Sex^a^			
Female	57 (71%)	7 (64%)	64 (70%)
Male	23 (29%)	4 (36%)	27 (30%)
Presence of liver metastasis	14 (18%)	3 (27%)	17 (19%)
PD-L1 expression			
<1%	24 (30%)	7 (64%)	31 (34%)
1–49%	22 (28%)	1 (9%)	23 (25%)
≥50%	25 (31%)	3 (27%)	31 (28%)
Not available	9 (11%)	0	9 (10%)
Anti-PD-(L)1 as last line of treatment before sotorasib	51 (64%)	N/A	N/A
Type of anti-PD-(L)1		N/A	N/A
Pembrolizumab^b^	58 (73%)		
200 mg Q3W	52 (65%)		
400 mg Q6W	6 (8%)		
Nivolumab	11 (14%)		
240 mg Q2W	5 (6%)		
480 mg Q4W	6 (8%)		
Durvalumab	9 (11%)		
Atezolizumab	2 (3%)		
Time on anti-PD-(L)1 (months), median (IQR)	6.7 (2.9–11.3)	N/A	N/A
Time between anti-PD-(L)1 and start sotorasib		N/A	N/A
Median, days (IQR)	62 (45–258)		
≤6 weeks	18 (23%)		
6–12 weeks	24 (30%)		
≥12 weeks	38 (48%)		
Prior hepatotoxicity during anti-PD-(L)1	8 (10%)	N/A	N/A
Time on sotorasib (months), median (IQR)	3.5 (2.2–7.2)	4.2 (2.7–14.0)	3.7 (2.6–7.0)
Starting dose sotorasib^c^			
960 mg	77 (96%)	11 (100%)	88 (97%)
480 mg	3 (4%)	0	3 (3%)

a Self-reported by patient.

b Monotherapy or in combination with platinum-based chemotherapy.

c At the treating physician's discretion patients could be started on a lower dose to minimize the risk of toxicities. Percentages may not add up to 100% due to rounding. IQR, interquartile range; PD-(L)1, programmed death (ligand)-1; QxW, every x weeks; N/A, not applicable.

### Hepatotoxicity

A total of 59 patients (65%) experienced hepatotoxicity during sotorasib, of whom 28 patients (31%) experienced severe hepatotoxicity. Among these 28 patients, five were hospitalized during the severe hepatotoxicity, of whom four patients presented with additional clinical problems that warranted hospitalization. Biliary tract obstruction was ruled out by ultrasound in seven cases, while viral causes of hepatitis were ruled out in eight cases, and auto-immune hepatitis was ruled out in three cases. None of the patients were concurrently using other medications known for a high risk of drug-induced liver injury. No patients underwent liver biopsies. Median time to first ALT/AST/GGT/ALP increase was 28 days (IQR 13–58), median time to grade ≥3 increase was 54 days (IQR 35–81). No patients had grade ≥3 bilirubin increase.

Prior anti-PD-(L)1 was not associated with a significantly higher incidence of any-grade hepatotoxicity compared to no prior anti-PD-(L)1 treatment (68% versus 73%, *p* = 0.74, Fisher’s Exact, OR 0.7 [95% CI 0.2–2.7]), but was associated with a significantly higher incidence of severe hepatotoxicity (35% versus 0%, *p* = 0.016, Fisher’s Exact, OR 12.5 [95% CI 0.7–219.8]). There was no statistically significant difference in incidence of severe hepatotoxicity between the different anti-PD-(L)1 regimens (22 out of 58 patients for pembrolizumab, five out of 11 for nivolumab, zero out of nine for durvalumab and one out of two for atezolizumab, *p* = 0.12, Chi Square). Patients who experienced immune-related hepatotoxicity during prior anti-PD-(L)1 treatment, had a higher incidence of severe hepatotoxicity during sotorasib than those who did not (75% versus 31%, *p* = 0.019, Fisher’s exact, OR 6.8 [95% CI 1.3–36.5]).

Of the patients who started sotorasib within six weeks of their last anti-PD-(L)1 course, 15 out of 18 patients (83%) experienced severe hepatotoxicity, versus eight out of 24 patients (33%) with an interval of six to 12 weeks between anti-PD-(L)1 and sotorasib (*n* = 24), and five out of 38 patients (13%) of the patients with an interval of more than 12 weeks (*n* = 38) (*p* < 0.0001, Chi Square test) (Table 2). The OR for the development of severe hepatotoxicity for ≤6 weeks between last anti-PD-(L)1 course and sotorasib initiation versus >6 weeks was 18.9 [95% CI 4.7–75.1].

**Table 2:** Worst grades ALT/AST/GGT/ALP increase, treatment consequences and days to development of severe hepatotoxicity during sotorasib treatment.

	No anti-PD-(L)1 *n* = 11	Prior anti-PD-(L)1 per interval between last anti-PD-(L)1 and sotorasib *n* = 80			
		≤6 weeks *n* = 18	6–12 weeks *n* = 24	≥12 weeks *n* = 38	*p*-value^b^
Prior immune-related hepatitis	N/A	2 (11%)	1 (4%)	5 (13%)	0.51
Any-grade hepatotoxicity	8 (73%)	17 (94%)	18 (75%)	16 (42%)	0.00028
Grade ≥ 3 hepatotoxicity	0	15 (83%)	8 (33%)	5 (13%)	<0.0001
Worst grade					0.00033
Grade 1	7 (64%)	2 (11%)	8 (33%)	6 (16%)	
Grade 2	1 (9%)	0 (0%)	2 (8%)	5 (13%)	
Grade 3	0 (0%)	10 (56%)	4 (17%)	4 (11%)	
Grade 4	0 (0%)	5 (28%)	4 (17%)	1 (3%)	
Treatment modification^a^	0 (0%)	15 (83%)	8 (33%)	3 (8%)	<0.0001
Interruption and reduction	0 (0%)	6 (33%)	5 (21%)	2 (5%)	
Treatment discontinuation	0 (0%)	9 (39%)	3 (13%)	1 (3%)	
Steroid treatment for hepatotoxicity during sotorasib	0 (0%)	9 (39%)	3 (13%)	2 (5%)	0.52
Time to grade ≥ 3 hepatotoxicity (days), median (IQR)	N/A	56 (32–78)	68 (43–89)	50 (30–61)	0.44^c^

Percentages may not add up to 100% due to rounding. Hepatotoxicity was defined as an increase in ALT, AST, GGT or ALP according to the Common Terminology Criteria for Adverse Events (CTCAE) Version 5. N/A, not applicable.

a Only including treatment modifications due to hepatotoxicity.

b Chi–Square Test, unless otherwise specified.

c Kruskal–Wallis Test.

### Management of severe hepatotoxicity

Among the patients with severe hepatotoxicity, treatment modification occurred in 26 out of 28 patients (93%). In 19 patients hepatotoxicity recurred after sotorasib interruption and dose reduction, leading to permanent treatment discontinuation in 13 patients. Nine patients were already on steroids prior to sotorasib initiation, of whom five were on an equivalent dose of 30 mg or higher of prednisone daily, primarily for arthritis (*n* = 3) or brain metastases (*n* = 2). Among these five patients, three developed severe hepatotoxicity. Additionally, one patient was on 40 mg of prednisone daily due to sotorasib-related colitis when they developed severe hepatotoxicity. Of the patients with severe hepatotoxicity (*n* = 28), fourteen received steroid treatment at the discretion of the treating physician at time of severe hepatotoxicity. Ten patients were started on 30 mg or higher of prednisone daily, typically for one week and then tapered by 10 mg per week. Nine of these patients experienced recurrence of hepatotoxicity upon the reintroduction of sotorasib, leading to treatment discontinuation in six patients. Four patients were started on an equivalent of less than 30 mg prednisone daily simultaneously with the reintroduction of sotorasib. Three of them experienced recurrence of hepatotoxicity, resulting in treatment discontinuation.

### Clinical outcomes

ORR was similar in patients with an interval between anti-PD-(L)1 treatment and sotorasib of ≤6 weeks and >6 weeks (33% [95% CI 16.1–56.4] versus 31% [95% CI 20.5–43.0], respectively, *p* = 0.83, Chi Square). Median PFS (5.3 months [95% CI 3.7–6.9] versus 4.8 months [95% CI 4.4–5.2], respectively, HR 1.01 [95% CI 0.54–1.89], *p* = 0.98, log-rank) and median OS (9.0 months [95% CI 5.7–12.4] versus 8.2 months [95% CI 5.9–10.4], respectively, HR 0.84 [95% CI 0.41–1.75], *p* = 0.64, log-rank) were also similar between groups. In patients who experienced severe hepatotoxicity, ORR was 36% (95% CI 20.6–54.2) versus 29% (95% CI 18.8–40.8) in those who did not (*p* = 0.50, Chi Square). In PFS analysis, 31 out of 91 cases (34%) were censored because of treatment discontinuation due to toxicity (*n* = 10), ongoing treatment at data cut-off (*n* = 20) or loss to follow up (*n* = 1). In OS analysis, 41 out of 91 cases (45%) were censored. Median PFS was 4.8 months (95% CI 4.3–5.3) in patients with severe hepatotoxicity versus 4.7 months (95% CI 3.9–5.5) in patients without severe toxicity (HR 1.12 [95% CI 0.63–2.0], *p* = 0.70, log-rank). Corresponding median OS was 9.0 months (95% CI 7.0–11.1) versus 8.5 months (95% CI 4.3–12.8), respectively (HR 0.91 [95% CI 0.49–1.70], *p* = 0.77, log-rank).

### Sotorasib concentrations

**Fig. 1:**
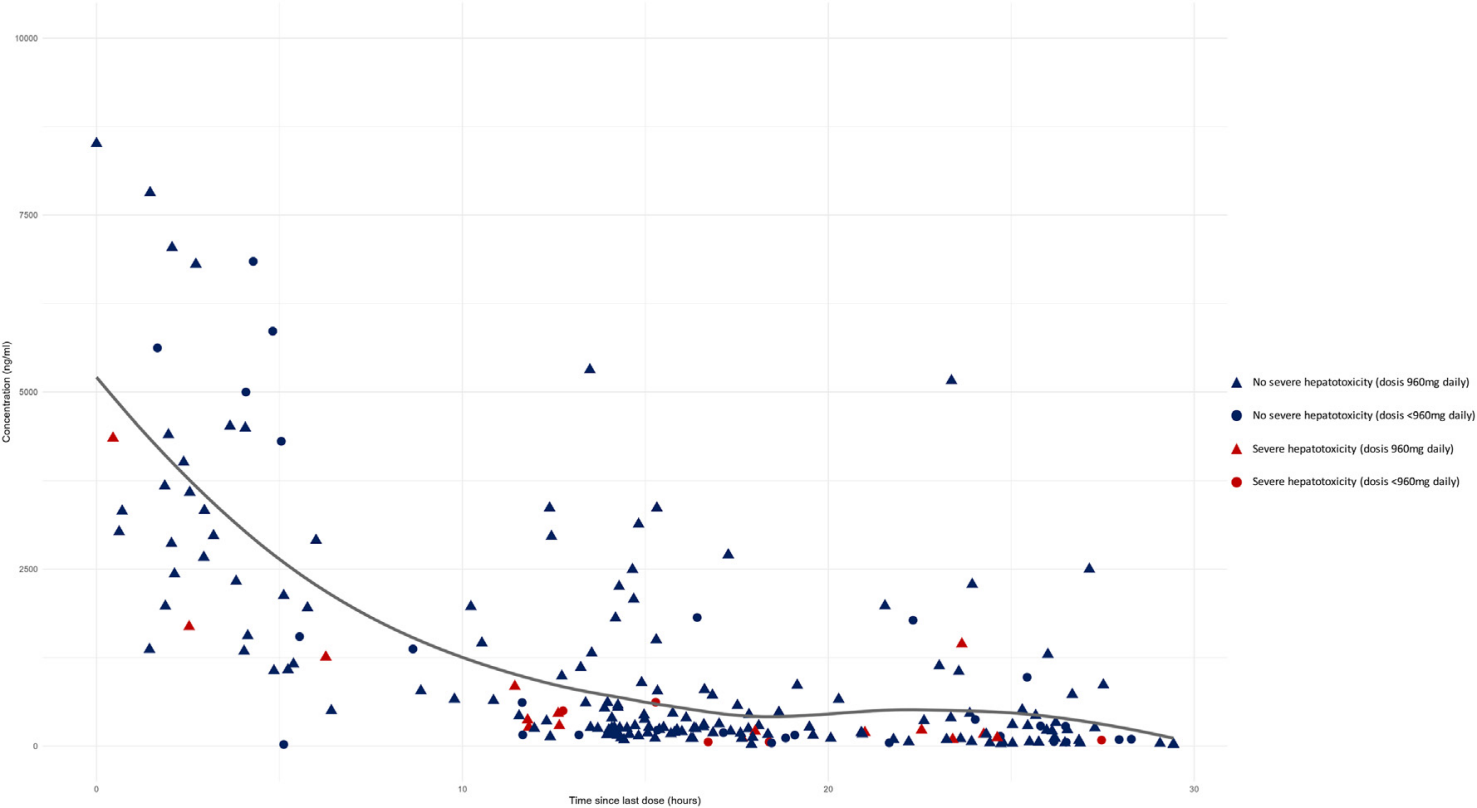
Concentration–time curve of all analysed plasma samples. Patients were asked the date and time of the last intake of sotorasib. This was used to calculate the time (hours) after intake (x-axis). The curve was fitted with the Loess method.

A total of 230 plasma samples of 81 patients were analysed for sotorasib concentrations. For statistical comparison of median C_trough_ between patients with and without severe hepatotoxicity, a total of 130 samples from 10 patients with severe hepatotoxicity and 44 patients without severe hepatotoxicity (range 1–12 samples per patient) were analysed. Median C_trough_ was 106 ng/mL in patients with severe hepatotoxicity versus 126 ng/mL in patients without severe hepatotoxicity (*p* = 0.16, Mann–Whitney U Test). For statistical comparison of median C_trough_ between samples that were collected during administration of a daily dose of 960 mg and lower doses, 134 samples were analysed. Median C_trough_ was 99 ng/mL for 960 mg and 109 ng/mL for lower doses (*p* = 0.99, Mann–Whitney U Test). Fig. 1 illustrates the concentration–time curve of all the analysed plasma samples (*n* = 230) and the relationship between severe hepatotoxicity and administered dose. The concentration–time curve with a log(10) scale is shown in Supplementary Fig. S1.

### Anti-PD-1 concentrations

Anti-PD-1 plasma concentration analysis was possible in 57 out of 58 cases who had previously received pembrolizumab, and in 10 out of 11 cases who had previously received nivolumab. Treatment time on pembrolizumab or nivolumab was not correlated with their respective plasma concentrations (*p* = 0.86 and *p* = 0.80, respectively, Spearman’s rho). In the patients who had received pembrolizumab, those who developed severe hepatotoxicity (*n* = 21) had much higher mean plasma concentrations of pembrolizumab than those who did not develop severe hepatotoxicity (*n* = 36) (25.6 versus 6.1 μg/mL, *p* < 0.0001, Student’s T-test). All plasma samples that were taken more than 132 days after last pembrolizumab course (*n* = 17) were below the detection threshold of 2.00 μg/mL. In the patients who had received nivolumab, the median plasma concentration was also higher in those who developed severe hepatotoxicity (*n* = 4) compared to those who did not (*n* = 6) (14.5 versus 7.2 μg/mL), although this did not reach statistical significance due to the small sample size (*p* = 0.61, Mann–Whitney U Test).

**Fig. 2:**
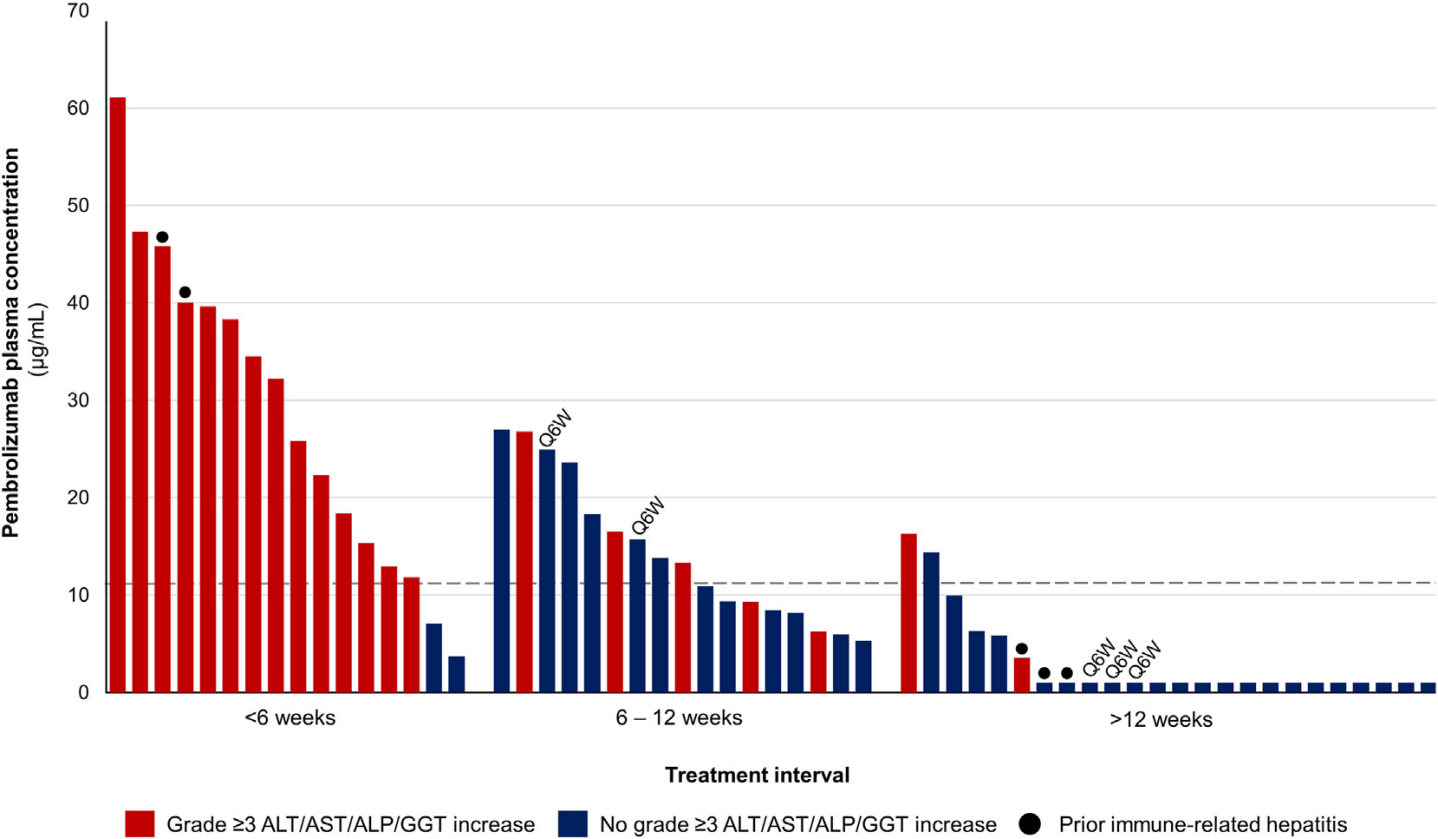
Plasma concentrations of pembrolizumab grouped per time interval between the last course of anti-PD-1 treatment and sotorasib initiation. Each bar represents an individual patient. Patients who received pembrolizumab 400 mg every 6 weeks are indicated with Q6W, the remaining patients received 200 mg every 3 weeks. The dashed horizontal line represents the cut-off value of 11.4 μg/mL that was determined in ROC curve analysis. PD-1, programmed death-1. ROC, Receiver Operating Characteristic.

**Fig. 3:**
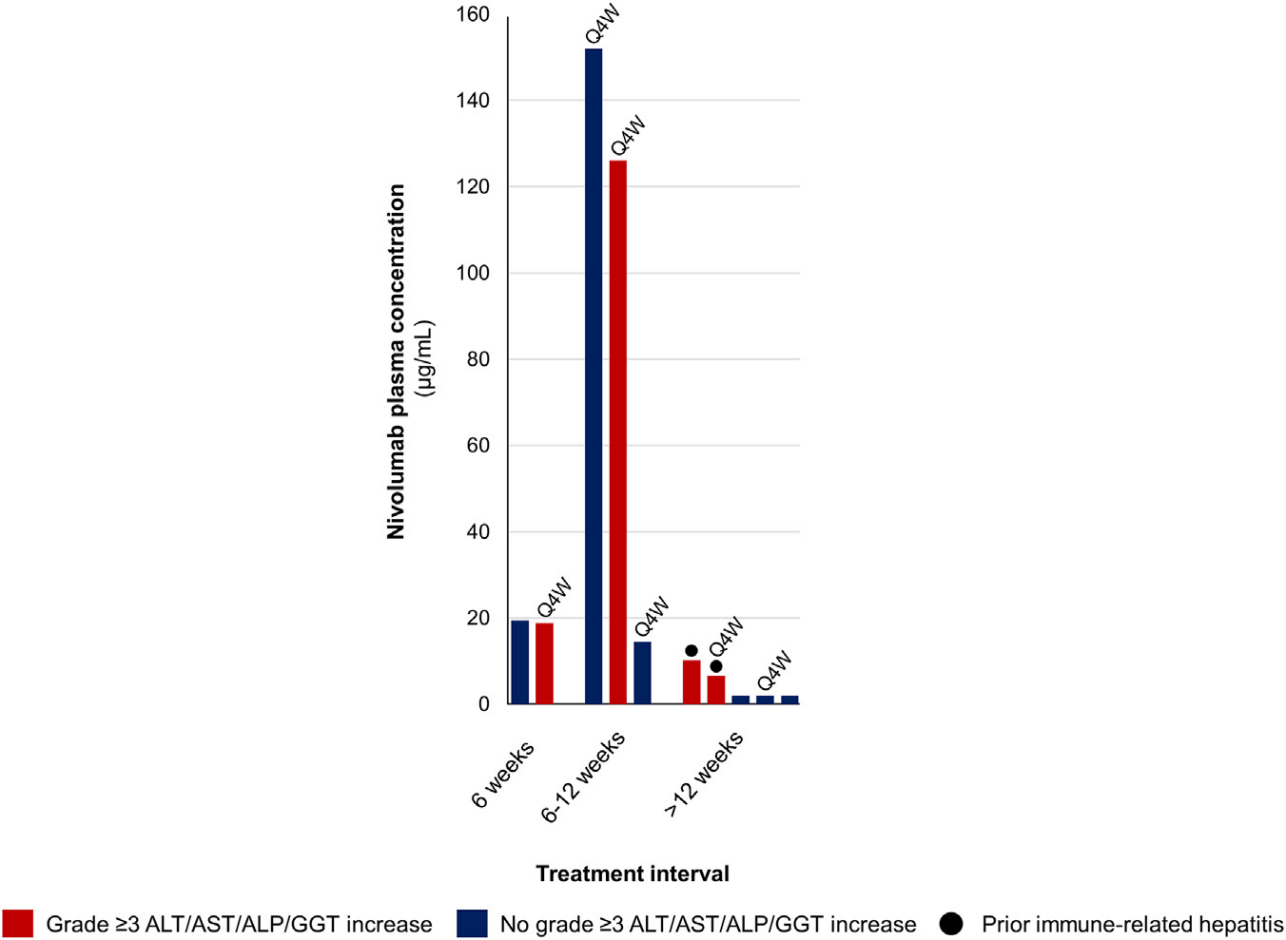
Plasma concentrations of pembrolizumab grouped per time interval between the last course of anti-PD-1 treatment and sotorasib initiation. Each bar represents an individual patient. Patients who received nivolumab 480 mg every 4 weeks are indicated with Q4W, the remaining patients received 240 mg every 2 weeks. PD-1, programmed death-1.

In ROC curve analysis within the analysed pembrolizumab population (*n* = 57), the area under the curve was 88.9% (95% CI 80.6–97.1) (Supplementary Fig. S2). The optimal cut-off value to predict the development of severe hepatotoxicity was set at 11.4 μg/mL, corresponding to a sensitivity of 86% (95% CI 64.5–95.9) and a specificity of 81% (95% CI 64.7–90.6) with a positive predictive value of 72% (95% CI 52.2–85.9) and a negative predictive value of 91% (95% CI 75.0–97.5). The incidence of severe hepatotoxicity was eight-fold higher in the ≥11.4 μg/mL group than in the <11.4 μg/mL group (72% versus 9%, OR 24.86 [95% CI 5.69–108.63], *p* < 0.0001, Chi Square) (Fig. 2). Three cases in the <11.4 μg/mL group developed severe hepatotoxicity, one of which had experienced a grade 3 immune-related hepatitis during prior pembrolizumab treatment. The plasma concentrations of nivolumab in relation to the occurrence of severe hepatotoxicity are visualized in Fig. 3, but not statistically tested due to the limited sample size.

### Univariate analysis

Univariate logistic regression analysis showed a significant association of patient age (≥65 versus <65 years) (OR 0.28 [95% CI 0.11–0.76]), prior immune-related hepatotoxicity (OR 6.82 [95% CI 1.28–36.48]), a treatment interval of less than 6 weeks between the last anti-PD-(L)1 treatment course and sotorasib initiation (OR 18.85 [95% CI 4.73–75.08]), and median pembrolizumab plasma concentration ((≥11.4 versus <11.4 μg/mL) (OR 24.86 [95% CI 5.69–108.63])), with the development of severe hepatotoxicity during sotorasib treatment (Table 3). No significant association was found for any of the other variables. Due to the modest sample size, subsequent multivariable analysis was not performed.

**Table 3:** Univariate logistic regression analysis on the association of investigated variables with the occurrence of grade ≥3 hepatotoxicity during sotorasib treatment.

Variable	Univariate analysis	*p*-value
	OR (95% CI)	
Sex (female versus male)	1.40 (0.51–3.82)	0.52
Age (≥65 years versus <65 years)	0.28 (0.11–0.76)	0.013
Liver metastasis (yes versus no)	0.25 (0.05–1.16)	0.076
Prior immune-related hepatotoxicity (yes versus no)	6.82 (1.28–36.48)	0.025
Anti-PD-(L)1 time on treatment (months, continuous scale)	1.01 (0.95–1.08)	0.71
Anti-PD-(L)1 interval (≤6 weeks versus >6 weeks)	18.85 (4.73–75.08)	<0.0001
Sotorasib time on treatment (months, continuous scale)	0.93 (0.84–1.03)	0.17
Median C_trough_ sotorasib (≥122 ng/mL versus <122 ng/mL)	0.82 (0.23–2.85)	0.75
Median pembrolizumab concentration (≥11.4 μg/mL versus <11.4 μg/mL)	24.86 (5.69–108.63)	<0.0001

OR, odds ratio. CI, confidence interval. PD-(L)1, programmed death-(ligand) 1. C_trough_, trough concentration.

## Discussion

This study showed a significant association between prior anti-PD-(L)1 treatment and anti-PD-1 plasma concentrations, and severe hepatotoxicity during sotorasib treatment. Importantly, our findings suggest that the timing of sotorasib initiation after anti-PD-(L)1 is a crucial determinant of the likelihood of severe hepatotoxicity during sotorasib treatment, irrespective of systemic sotorasib exposure.

The majority of patients (83%) who started sotorasib within 6 weeks after receiving anti-PD-(L)1 treatment experienced severe hepatotoxicity, leading to permanent discontinuation of sotorasib in 39% of them. In patients with an interval of 6–12 weeks between anti-PD-(L)1 and sotorasib, only 33% developed severe hepatotoxicity, and in those with an interval of more than 12 weeks, the incidence was even lower (13%). In comparison, a recent retrospective cohort study found that only 7 out of 181 patients with advanced NSCLC (3.9%) receiving immune checkpoint blockade monotherapy experienced grade ≥3 ALT or AST increase.^14^ This suggests that the cases of hepatotoxicity in our cohort were unlikely to be caused by delayed toxicity from anti-PD-(L)1 treatment alone. Additionally, we identified a history of immune-related hepatotoxicity as a risk factor for the development of severe hepatotoxicity during sotorasib. A prior study showed that hepatitis was among the immune-related adverse events with high recurrence rates in patients who received a rechallenge of immunotherapy after a prior immune-related adverse event, which could potentially explain why we found this to be a risk factor for severe hepatotoxicity during sotorasib.^15^ These findings also raise the question whether patients in which the expected benefit of anti-PD-(L)1 treatment (e.g., patients with low or absent PD-L1 expression or with *STK11* or *KEAP1* co-mutations) is limited,^16^ anti-PD-(L)1 treatment should not be saved for after treatment with sotorasib. Currently, the CodeBreaK201 is investigating sotorasib 960 mg daily versus 240 mg daily as first-line treatment in patients with *KRAS*^G12C^-mutated NSCLC with a PD-L1 tumour proportion score of <1% and/or presence of a *STK11* mutation (NCT04933695). The outcomes of this study could potentially aid in answering this question. Next, following the rationale of the CodeBreaK 100/101 of combining immunotherapy and sotorasib, and considering the long half-life of anti-PD-(L)1 agents,^17^, ^18^, ^19^, ^20^, ^21^, ^22^ it is possible that sequential sotorasib treatment after recent anti-PD-(L)1 elicits improved treatment responses. However, our results suggest similar clinical outcomes in patients with a ≤6 week interval between sequential treatments and those with a >6 week interval.

There was no significant difference in the systemic sotorasib trough concentration between patients who experienced severe hepatotoxicity and those who did not. This finding further emphasizes that prior anti-PD-(L)1 treatment is likely the main factor determining the risk of hepatotoxicity, irrespective of sotorasib exposure. Moreover, no significant difference was observed in sotorasib trough concentrations between patients receiving the maximum approved dose of 960 mg daily and those on lower doses, consistent with previous pharmacokinetic studies of sotorasib at various doses.^11^^,^^23^ However, it remains to be determined whether sotorasib-associated toxicity is associated with trough levels or maximum concentrations. Nonetheless, there is some evidence suggesting that using lower doses may potentially reduce the risk of hepatotoxicity as sotorasib is metabolized in the liver, and thus higher maximum concentrations might lead to accumulation of sotorasib metabolite in the liver.^24^^,^^25^ This also underscores the importance of the ongoing debate on the utility of maximum tolerated doses in targeted therapies.^26^^,^^27^

Lastly, we found that in patients who had received pembrolizumab prior to sotorasib, pembrolizumab plasma concentrations were significantly higher in those who developed severe hepatotoxicity compared to those who did not. Although plasma drug concentrations decrease with time, prior studies have shown that several patient-related factors can significantly influence immunotherapy drug pharmacokinetics and clearance.^21^^,^^22^ Consequently, the plasma concentrations of anti-PD-(L)1 agents at time of sotorasib initiation may vary substantially among patients, regardless of the time since their last anti-PD-(L)1 course. In our cohort we indeed found that pembrolizumab plasma concentrations varied between patients regardless of time since last pembrolizumab infusion. This variation could hold crucial clinical importance as the majority of patients who are eligible for sotorasib, have progressed on at least one line of prior systemic treatment. Thus, these patients often have a substantial tumour burden and delaying the initiation of sotorasib, unless absolutely necessary to minimize toxicity risks, may not be desirable. Our results suggest that pembrolizumab plasma concentrations could potentially aid in identifying patients who are at higher risk of severe hepatotoxicity during sotorasib treatment. However, our determined cut-off value of 11.4 μg/mL should be validated in a larger cohort. Additionally, the potential risk and benefits of the timing of sotorasib initiation after prior anti-PD-(L)1 treatment should be assessed on an individual patient basis.

The exact underlying mechanism of sotorasib-induced liver damage remains unclear. Prior studies have shown that kinase inhibitors can induce immune-related toxicities in patients previously treated with anti-PD-(L)1 therapy.^28^, ^29^, ^30^, ^31^ Although sotorasib is not a tyrosine kinase inhibitor, it does seem that targeted therapies can trigger immune responses in immunotherapy-exposed patients. Preclinical studies have suggested that sotorasib may create a pro-inflammatory tumour microenvironment with increased CD8-positive T-cell infiltration.^32^ Although this presents a treatment opportunity to combine sotorasib with immunotherapy, it may also contribute to the development of immune-related toxicities. Therefore, in the case of severe hepatotoxicity, clinicians should also consider corticosteroids or other immunosuppressive agents in addition to treatment interruption. However, the optimal management of severe hepatotoxicity in this patient population remains challenging, as demonstrated by the high discontinuation rate in our cohort despite steroid treatment. Adagrasib, another small molecule KRAS^G12C^-inhibitor, has thus far demonstrated less hepatotoxicity when used in combination with immunotherapy compared to sotorasib.^33^ This suggests that the mechanism of hepatotoxicity may be drug-specific rather than class-specific. However, it is important to note that the currently available safety data on adagrasib is more limited compared to that of sotorasib.

Our study has some limitations that should be considered. Firstly, we did not perform liver biopsies which limits our ability to draw definitive conclusions regarding its aetiology. Furthermore, serological or molecular testing for viral or autoimmune diseases was not performed in all cases. However, given the setting and the time-dependent association with sotorasib, it is unlikely that testing for viral or autoimmune causes would have altered our findings. Secondly, although this is the largest real-world prospective cohort of sequential anti-PD-(L)1 and sotorasib reported so far, the sample size was modest. Therefore, further statistical analysis, such as multivariable analysis, was not performed. Consequently, we were not able to adjust for confounders and confirm whether the variables associated with risk of severe hepatotoxicity are independent risk factors. Furthermore, the limited sample size introduces the potential for sparse data bias, evident in the high ORs and wide 95% CIs we observed in univariate logistic regression. Consequently, the reported effect sizes may potentially be overestimated. Finally, the equation used to calculate sotorasib trough levels has not been validated for sotorasib, so these analyses should be considered exploratory. However, given the linear pharmacokinetics of sotorasib after the initial maximum concentration has been reached, it is probable that our equation provided a sufficient approximation of the trough concentrations. Despite these limitations, to the best of our knowledge, no other prospective studies have incorporated both anti-PD-1 and sotorasib plasma concentrations to provide a comprehensive clinical and pharmacokinetic analysis of the time-dependent relationship between sequential anti-PD-(L)1 and sotorasib treatment and the development of severe hepatotoxicity.

In conclusion, our study highlights that severe hepatotoxicity is a significant concern in patients receiving sequential anti-PD-(L)1 followed by sotorasib, especially in those with a short interval between treatments, a history of immune-related hepatotoxicity or higher pembrolizumab plasma concentrations. As no association with systemic sotorasib plasma concentrations was found, this further suggests that prior anti-PD-(L)1 is the major determinant of the risk of hepatotoxicity. Our results would suggest a minimum interval of six weeks between the last anti-PD-(L)1 course and sotorasib initiation to reduce the risk of hepatotoxicity. However, considering the limitations that are inherent to the limited sample size, these findings should be considered preliminary. Larger, randomized trials are needed to validate and better understand these associations.

## Contributors

S.M. Ernst: conceptualization, formal analysis, investigation, methodology, visualization, writing - original draft, writing - review & editing. M.M. Hofman: conceptualization, methodology, writing - review & editing. T.E. van der Horst: investigation, writing - review & editing. M.S. Paats: conceptualization, methodology, writing - review & editing. F.W.J. Heijboer: conceptualization, methodology, visualization, writing - review & editing. J.G.J.V. Aerts: conceptualization, methodology, writing - review & editing. D.W. Dumoulin: conceptualization, methodology, writing - review & editing. R. Cornelissen: conceptualization, methodology, writing - review & editing. J.H. von der Thusen: conceptualization, methodology, writing - review & editing. P. de Bruijn: data curation, formal analysis, investigation, writing - review & editing. E. Oomen-de Hoop: methodology, formal analysis, writing - review & editing. R.H.J. Mathijssen: conceptualization, methodology, supervision, writing - review & editing. S.L.W. Koolen: conceptualization, methodology, supervision, writing - review & editing. A.C. Dingemans: conceptualization, methodology, supervision, writing - review & editing. All authors had full access to all the data in the study. SE, MH and TvdH verified the clinical data; SE, PdB and SK verified the pharmacokinetic data. All authors have read and approved the final version of the manuscript.

## Data sharing statement

The data used and analysed in the present study are available from the corresponding author upon reasonable request.

## Declaration of interests

M.S. Paats reports receiving institutional fees from AstraZeneca, Bayer, Eli Lilly, Janssen, Novartis, Pfizer, Roche and Takeda; outside the current work. J.G.J.V. Aerts reports receiving advisory board and speakers fees from Eli Lilly, BMS, MSD, AstraZeneca, Bayer, Amphera and is a stock owner of Amphera; outside the current work. D.W. Dumoulin reports receiving consulting fees from BMS, MSD, Pfizer, Amgen and Roche; outside the current work. R.C. Cornelissen reports receiving advisory board and speakers fees from MSD, Janssen, Librerium and Spectrum; outside the current work. J.H. von der Thüsen reports receiving advisory board and speakers fees from Eli Lilly, BMS, MSD, AstraZeneca, Bayer, Janssen, Pfizer, Amgen, and institutional receipt of materials of Roche; outside the current work. R.H.J. Mathijssen reports receiving institutional fees for investigator-initiated trials from Astellas, Bayer, Boehringer-Ingelheim, Cristal Therapeutics, Novartis, Pamgene, Pfizer, Roche, Sanofi and Servier; outside the current work. S.L.W. Koolen reports receiving speakers fee from Promise Proteomics; outside the current work. A.C Dingemans reports receiving institutional fees from Roche, Eli Lilly, Boehringer Ingelheim, AstraZeneca, Janssen, Chiezi, Amgen, Pfizer, Bayer, Takeda, Pharmamar, Sanofi and Daiichi, and grants from the Dutch Cancer Society, HANARTH and Amgen paid to the institution; outside the current work; chair EORTC Lung Cancer group. All other authors report no disclosures.

## References

[1] Gandhi L., Rodríguez-Abreu D., Gadgeel S. (2018). Pembrolizumab plus chemotherapy in metastatic non-small-cell lung cancer. N Engl J Med.

[2] Greenhalgh J., Boland A., Bates V. (2021). First-line treatment of advanced epidermal growth factor receptor (EGFR) mutation positive non-squamous non-small cell lung cancer. Cochrane Database Syst Rev.

[3] Peters S., Camidge D.R., Shaw A.T. (2017). Alectinib versus crizotinib in untreated ALK-positive non-small-cell lung cancer. N Engl J Med.

[4] de Langen A.J., Johnson M.L., Mazieres J. (2023). Sotorasib versus docetaxel for previously treated non-small-cell lung cancer with KRAS(G12C) mutation: a randomised, open-label, phase 3 trial. Lancet.

[5] Dogan S., Shen R., Ang D.C. (2012). Molecular epidemiology of EGFR and KRAS mutations in 3,026 lung adenocarcinomas: higher susceptibility of women to smoking-related KRAS-mutant cancers. Clin Cancer Res.

[6] Chour A., Denis J., Mascaux C. (2023). Severe sotorasib-related hepatotoxicity and non-liver adverse events associated with sequential anti-PD(L)1 and sotorasib therapy in KRAS(G12C)-mutant lung cancer. J Thorac Oncol.

[7] Begum P., Goldin R.D., Possamai L.A., Popat S. (2021). Severe immune checkpoint inhibitor hepatitis in KRAS G12C-mutant NSCLC potentially triggered by sotorasib: case report. JTO Clin Res Rep.

[8] Desai A., Rakshit S., Bansal R. (2023). Time from immune checkpoint inhibitor to sotorasib use correlates with risk of hepatotoxicity in non-small cell lung cancer: a brief report. Cancer Treat Res Commun.

[9] Thummalapalli R., Bernstein E., Herzberg B. (2023). Clinical and genomic features of response and toxicity to sotorasib in a real-world cohort of patients with advanced KRAS G12C-mutant non-small cell lung cancer. JCO Precis Oncol.

[10] Li B.T., Falchook G.S., Durm G.A. (2022). OA03.06 - CodeBreaK 100/101: first report of safety/efficacy of sotorasib in combination with pembrolizumab or atezolizumab in advanced KRAS p.G12C NSCLC [abstract]. World Conf Lung Cancer.

[11] FDA Prescribing information LUMAKRAS (sotorasib) 2021. https://www.accessdata.fda.gov/drugsatfda_docs/label/2021/214665s000lbl.pdf.

[12] van Eerden R.A.G., Oomen-de Hoop E., Noordam A., Mathijssen R.H.J., Koolen S.L.W. (2021). Feasibility of extrapolating randomly taken plasma samples to trough levels for therapeutic drug monitoring purposes of small molecule kinase inhibitors. Pharmaceuticals.

[13] Marin C., Khoudour N., Millet A. (2021). Cross-validation of a multiplex LC-MS/MS method for assaying mAbs plasma levels in patients with cancer: a GPCO-UNICANCER study. Pharmaceuticals.

[14] Basak E.A., Vermeer N.S., de Joode K. (2022). Associations between patient and disease characteristics and severe adverse events during immune checkpoint inhibitor treatment: an observational study. Eur J Cancer.

[15] Dolladille C., Ederhy S., Sassier M. (2020). Immune checkpoint inhibitor rechallenge after immune-related adverse events in patients with cancer. JAMA Oncol.

[16] Alessi J.V., Elkrief A., Ricciuti B. (2023). Clinicopathologic and genomic factors impacting efficacy of first-line chemoimmunotherapy in advanced NSCLC. J Thorac Oncol.

[17] FDA Prescribing information KEYTRUDA (pembrolizumab) 2014. https://www.accessdata.fda.gov/drugsatfda_docs/label/2021/125514s096lbl.pdf.

[18] FDA Prescribing information OPDIVO (nivolumab) 2014. https://www.accessdata.fda.gov/drugsatfda_docs/label/2018/125554s058lbl.pdf.

[19] FDA Prescribing information TECENTRIQ (atezolizumab) 2016. https://www.accessdata.fda.gov/drugsatfda_docs/label/2021/761034s042lbl.pdf.

[20] FDA Prescribing information IMFINZI (durvalumab) 2017. https://www.accessdata.fda.gov/drugsatfda_docs/label/2022/761069s035lbl.pdf.

[21] Hurkmans D.P., Basak E.A., van Dijk T. (2019). A prospective cohort study on the pharmacokinetics of nivolumab in metastatic non-small cell lung cancer, melanoma, and renal cell cancer patients. J Immunother Cancer.

[22] Hurkmans D.P., Sassen S.D.T., de Joode K. (2021). Prospective real-world study on the pharmacokinetics of pembrolizumab in patients with solid tumors. J Immunother Cancer.

[23] (2020). Center for drug evaluation and research application number 214665Orig1s000. Drug approval package: lumakras. https://www.accessdata.fda.gov/drugsatfda_docs/nda/2021/214665Orig1s000TOC.cfm.

[24] Ivanov S.M., Lagunin A.A., Filimonov D.A., Poroikov V.V. (2022). Relationships between the structure and severe drug-induced liver injury for low, medium, and high doses of drugs. Chem Res Toxicol.

[25] Lammert C., Bjornsson E., Niklasson A., Chalasani N. (2010). Oral medications with significant hepatic metabolism at higher risk for hepatic adverse events. Hepatology.

[26] Ratain M.J., Tannock I.F., Lichter A.S. (2021). Dose optimization of sotorasib: is the US food and drug administration sending a message?. J Clin Oncol.

[27] Soulières D., Gelmon K.A. (2021). Sotorasib: is maximum tolerated dose really the issue at hand?. J Clin Oncol.

[28] Schoenfeld A.J., Arbour K.C., Rizvi H. (2019). Severe immune-related adverse events are common with sequential PD-(L)1 blockade and osimertinib. Ann Oncol.

[29] McCoach C.E., Rolfo C., Drilon A. (2022). Hypersensitivity reactions to selpercatinib treatment with or without prior immune checkpoint inhibitor therapy in patients with NSCLC in LIBRETTO-001. J Thorac Oncol.

[30] Lin J.J., Chin E., Yeap B.Y. (2019). Increased hepatotoxicity associated with sequential immune checkpoint inhibitor and crizotinib therapy in patients with non-small cell lung cancer. J Thorac Oncol.

[31] Oshima Y., Tanimoto T., Yuji K., Tojo A. (2018). EGFR-TKI-Associated interstitial pneumonitis in nivolumab-treated patients with non-small cell lung cancer. JAMA Oncol.

[32] Canon J., Rex K., Saiki A.Y. (2019). The clinical KRAS(G12C) inhibitor AMG 510 drives anti-tumour immunity. Nature.

[33] Jänne P.A., Smit E.F., de Marinis F. (2022). LBA4 Preliminary safety and efficacy of adagrasib with pembrolizumab in treatment-naive patients with advanced non-small cell lung cancer (NSCLC) harboring a KRASG12C mutation. Ann Oncol.

