# Supplementary figures and images for: Hepatotoxicity in patients with non-small cell lung cancer treated with sotorasib after prior immunotherapy: a comprehensive clinical and pharmacokinetic analysis
Source: eBioMedicine. 2024 Mar 19;102:105074. doi: 10.1016/j.ebiom.2024.105074 (PMC10960098; doi:10.1016/j.ebiom.2024.105074)

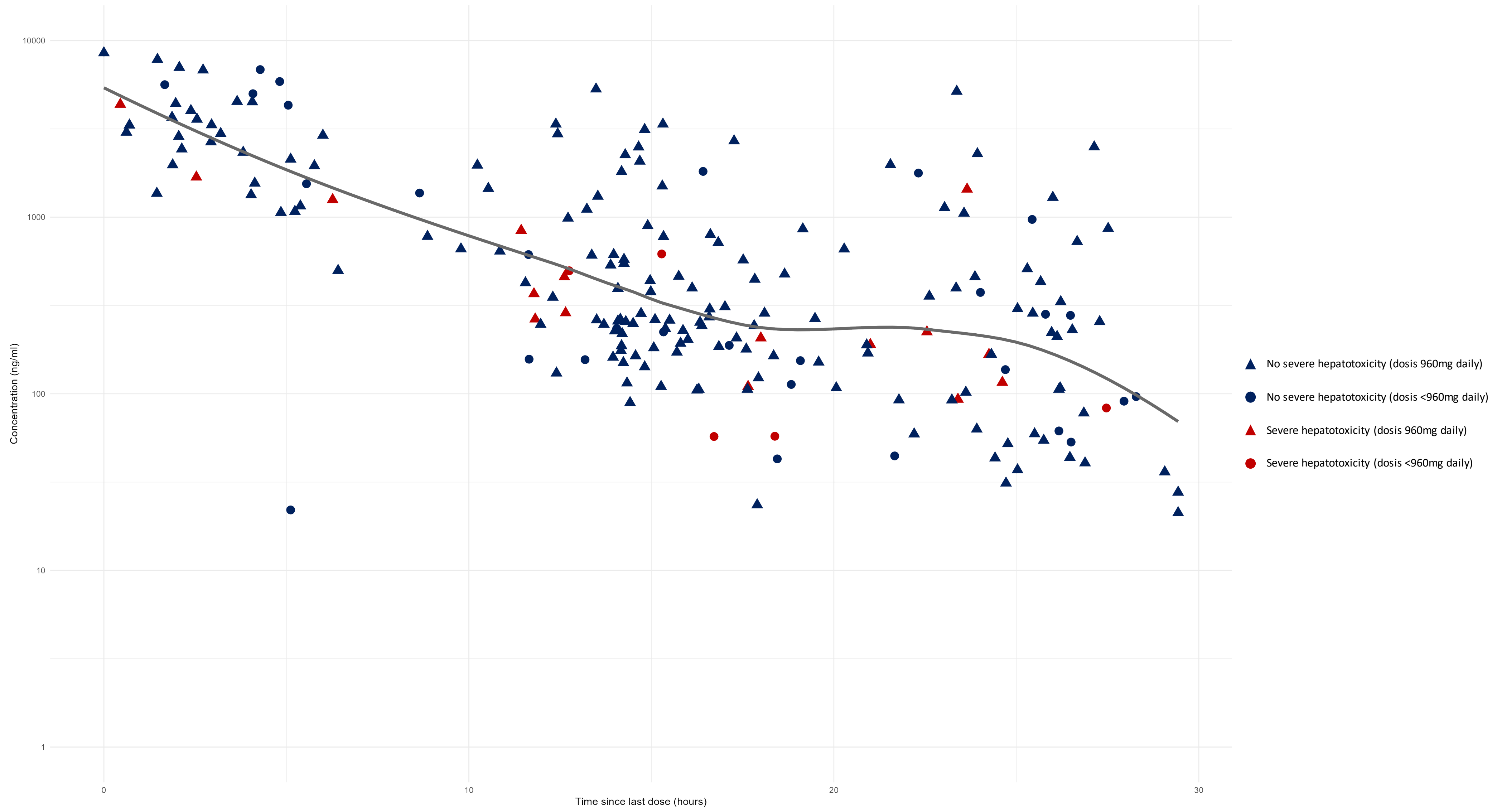

Supplement: Supplementary Fig. S1 [file mmc1.pdf]

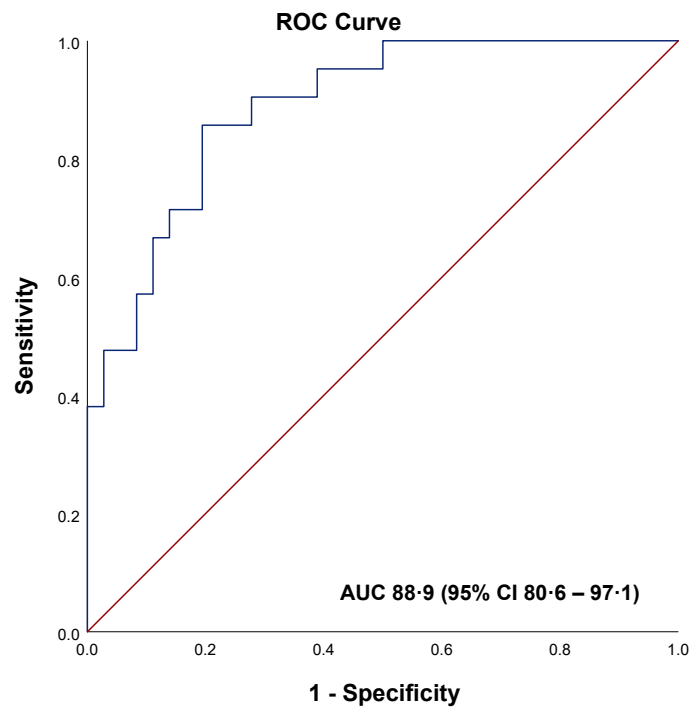

Supplement: Supplementary Fig. S2 [file mmc2.pdf]
