# Supplementary material for: Hepatotoxicity in patients with non-small cell lung cancer treated with sotorasib after prior immunotherapy: a comprehensive clinical and pharmacokinetic analysis
Source: eBioMedicine. 2024 Mar 19;102:105074. doi: 10.1016/j.ebiom.2024.105074 (PMC10960098; doi:10.1016/j.ebiom.2024.105074)
Supplement: Supplementary Figure legends [file mmc3.docx]

**Figure legends for supplementary figures for “Hepatotoxicity in patients with non-small cell lung cancer treated with sotorasib after prior immunotherapy: a comprehensive clinical and pharmacokinetic analysis.”** by Sophie M. Ernst, Maaike M. Hofman, Tessa E. van der Horst, Marthe S. Paats, Frank W.J. Heijboer, Joachim G.J.V. Aerts, Daphne W. Dumoulin, Robin Cornelissen, Jan H. von der Thüsen, Peter de Bruijn, Esther Oomen-de Hoop, Ron H.J. Mathijssen, Stijn L.W. Koolen, Anne-Marie C. Dingemans.

**Supplementary Figure 1.** Concentration-time curve of all analysed plasma samples with a Log(10) scale. Patients were asked the date and time of the last intake of sotorasib. This was used to calculate the time (hours) after intake (x-axis). The curve was fitted with the Loess method.

**Supplementary Figure 2**. Receiver Operating Characteristic (ROC) curve to explore the optimal cut-off value of pembrolizumab plasma concentrations for prediction of severe hepatotoxicity. AUC, area under the curve. CI, confidence interval.
